# Simultaneous subcortical and cortical electrophysiological recordings of spectro-temporal processing in humans

**DOI:** 10.3389/fneur.2022.928158

**Published:** 2022-08-03

**Authors:** Axelle Calcus, Jaime A. Undurraga, Deborah Vickers

**Affiliations:** ^1^Department of Speech, Hearing and Phonetic Sciences, University College London, London, United Kingdom; ^2^Laboratoire des Systèmes Perceptifs, Département d'Etudes Cognitives, Ecole Normale Supérieure, PSL University, CNRS, Paris, France; ^3^Center for Research in Cognitive Neuroscience, Université Libre de Bruxelles (ULB), Brussels, Belgium; ^4^Department of Linguistics, Macquarie University, Sydney, NSW, Australia; ^5^Interacoustics Research Unit, Technical University of Denmark, Lyngby, Denmark; ^6^SOUND Lab, Cambridge Hearing Group, Department of Clinical Neurosciences, Herchel Smith Building for Brain and Mind Sciences, Cambridge, United Kingdom

**Keywords:** auditory change complex, frequency following response (FFR), cortical auditory evoked potential (CAEP), brainstem, auditory processing (AP)

## Abstract

Objective assessment of auditory discrimination has often been measured using the Auditory Change Complex (ACC), which is a cortically generated potential elicited by a change occurring within an ongoing, long-duration auditory stimulus. In cochlear implant users, the electrically-evoked ACC has been used to measure electrode discrimination by changing the stimulating electrode during stimulus presentation. In addition to this cortical component, subcortical measures provide further information about early auditory processing in both normal hearing listeners and cochlear implant users. In particular, the frequency-following response (FFR) is thought to reflect the auditory encoding at the level of the brainstem. Interestingly, recent research suggests that it is possible to simultaneously measure both subcortical and cortical physiological activity. The aim of this research was twofold: first, to understand the scope for simultaneously recording both the FFR (subcortical) and ACC (cortical) responses in normal hearing adults. Second, to determine the best recording parameters for optimizing the simultaneous capture of both responses with clinical applications in mind. Electrophysiological responses were recorded in 10 normally-hearing adults while they listened to 16-second-long pure tone sequences. The carrier frequency of these sequences was either steady or alternating periodically throughout the sequence, generating an ACC response to each alternation—the alternating ACC paradigm. In the “alternating” sequences, both the alternating rate and the carrier frequency varied parametrically. We investigated three alternating rates (1, 2.5, and 6.5 Hz) and seven frequency pairs covering the low-, mid-, and high-frequency range, including narrow and wide frequency separations. Our results indicate that both the slowest (1 Hz) and medium (2.5 Hz) alternation rates led to significant FFR and ACC responses in most frequency ranges tested. Low carrier frequencies led to larger FFR amplitudes, larger P1 amplitudes, and N1-P2 amplitude difference at slow alternation rates. No significant relationship was found between subcortical and cortical response amplitudes, in line with different generators and processing levels across the auditory pathway. Overall, the alternating ACC paradigm can be used to measure sub-cortical and cortical responses as indicators of auditory early neural encoding (FFR) and sound discrimination (ACC) in the pathway, and these are best obtained at slow alternation rates (1 Hz) in the low-frequency range (300–1200 Hz).

## Introduction

Auditory evoked potentials are electrophysiological responses providing information on underlying neurophysiological function of structures in the auditory pathways. They are useful in audiological diagnostic assessment and for populations who cannot provide reliable responses to sounds. Electrophysiological responses are routinely used to explore the viability of different stages of the auditory pathway, from otoacoustic emissions, recording responses from the organ of Corti, through to cortical auditory evoked potentials, showing responsiveness of higher brain centers [e.g., (1–3)]. However, measurements can be time consuming particularly if responses to multiple stimulus parameters are required, for example, when recording responses to different sound frequencies. Measurement of responses at different stages in the auditory pathway allow for identification of site of lesion or loci of sound transmission difficulties for individuals with atypical sound processing abilities. The best approach to understanding sound processing at different stages of the auditory pathway is to measure concurrent responses at different sites.

Knebel et al. (4) have suggested that the combination of speech auditory brainstem responses (ABRs) and cortical responses to the same stimuli can be used to understand the inter-relationship between the generators of the different potentials and also the interaction between different brain regions. Musacchia et al. (5) recorded simultaneous speech ABRs and cortical onset responses (ORs) to /da/ stimuli to determine if musicians compared to non-musicians exhibited differences in ABRs and associated cortical ORs. They found that stronger ABRs to periodicity was associated with shorter latency of the OR and that musicians showed larger ABR amplitudes and shorter OR latencies than non-musicians.

Krishnan et al. (6) reported an approach for simultaneously acquiring the brainstem frequency following response (FFR) and cortical evoked pitch responses. The FFR is a sustained response evoked by the neurons in the brainstem able to track, on a cycle-by-cycle basis, the frequency of the periodic stimuli—phase locking. Pitch salience was varied by adapting the number of stimulus periodicity in an iterated rippled noise. The cortical responses to pitch were measured for stimulus onset (OR) and in response to a change in the pitch salience of the stimulus [auditory change complex, ACC (7)]. The ACC is a cortical response evoked by a change in an ongoing stimulus, with a fronto-central topographic distribution when referenced to the mastoid (7, 8). Morphologically, the ACC is characterized by a series of peaks usually within 50 and 250 ms after the stimulus onset – P1-N1-P2 response – and is measured using EEG electrodes typically placed in fronto-central regions. The latency, amplitude and morphology of the peaks (P1, P2) and trough (N1) are used as indicators of neural synchrony and maturation of the auditory pathways. Contrary to the OR, in which response characteristics have not been associated with pitch salience, the magnitude and latency of the ACC show a clear relation with pitch perception. For example, Mathew et al. (9) observed strong associations between the ACC and the ability to discriminate between stimulating electrodes in cochlear implant (CI) users. There is evidence that ACC responses to change in stimulus characteristics relate to speech perception abilities: Han and Dimitrijevic (10) showed a relationship between the N1 latency for the ACC to modulation detection and speech perception. However, behavioral discrimination seems to be best predicted by combining both subcortical (brainstem FFRs) and cortical (ACC) responses (6) to improve understanding of the processing in different auditory regions.

This approach for simultaneous measurement of the brainstem FFR and the ACC is of interest here. By means of a modified ACC paradigm, in which the fundamental frequency (F0) of an otherwise continuous stimulus, is periodically alternated—the alternating ACC (8, 11) - we investigate spectro-temporal processing in subcortical and cortical regions. The goals of the current research were to determine if brainstem FFRs and cortical ACC responses could be evoked and recorded simultaneously to periodic frequency alternations in a stimulus, allowing multiple measurements across the auditory pathway to investigate F0 processing. We varied parameters to understand the optimal approach for maximizing responses. This research is directed at developing electrophysiological measures that can help to understand perceptual capabilities in normal hearing, hearing impairment, and listening with a CI. In particular, we aim to develop electrophysiological paradigms that can be efficiently used to objectively measure discrimination and temporal processing abilities, hence allowing for identification of spectral regions where signal transmission/processing might be impaired. Such measures can also be used to evaluate phase locking and adaptation in the auditory system (8).

Here, we investigate subcortical and cortical functional integrity to periodic changes in F0 occurring at several alternating rates. F0s were chosen to correspond to center frequencies of CI electrodes, ranging from 300 to 3,000 Hz, for future application with CI users (using Advanced Bionics frequency allocation table). Alternation rates varied from 1 to 6.5 Hz, hence being close to the syllabic rate. This paradigm aimed to identify the condition that would provide the most information in a minimum amount of time, in the objective of developing a reliable, fast clinical tool.

## Methods

### Participants

Ten young (21–27 years old, mean 23.66 years, 2 males) English speakers participated in this study. All participants had normal hearing defined as air-conducted pure-tone thresholds of 25 dB HL or better at octave frequencies from 0.25 to 8 kHz in both ears. None of the participants reported a history of neurological disorders. All participants provided written consent as approved by the UCL Research Ethics Committee (SHaPS-2018-DV-028) and were compensated for their time.

### Stimuli

**Figure 1:**
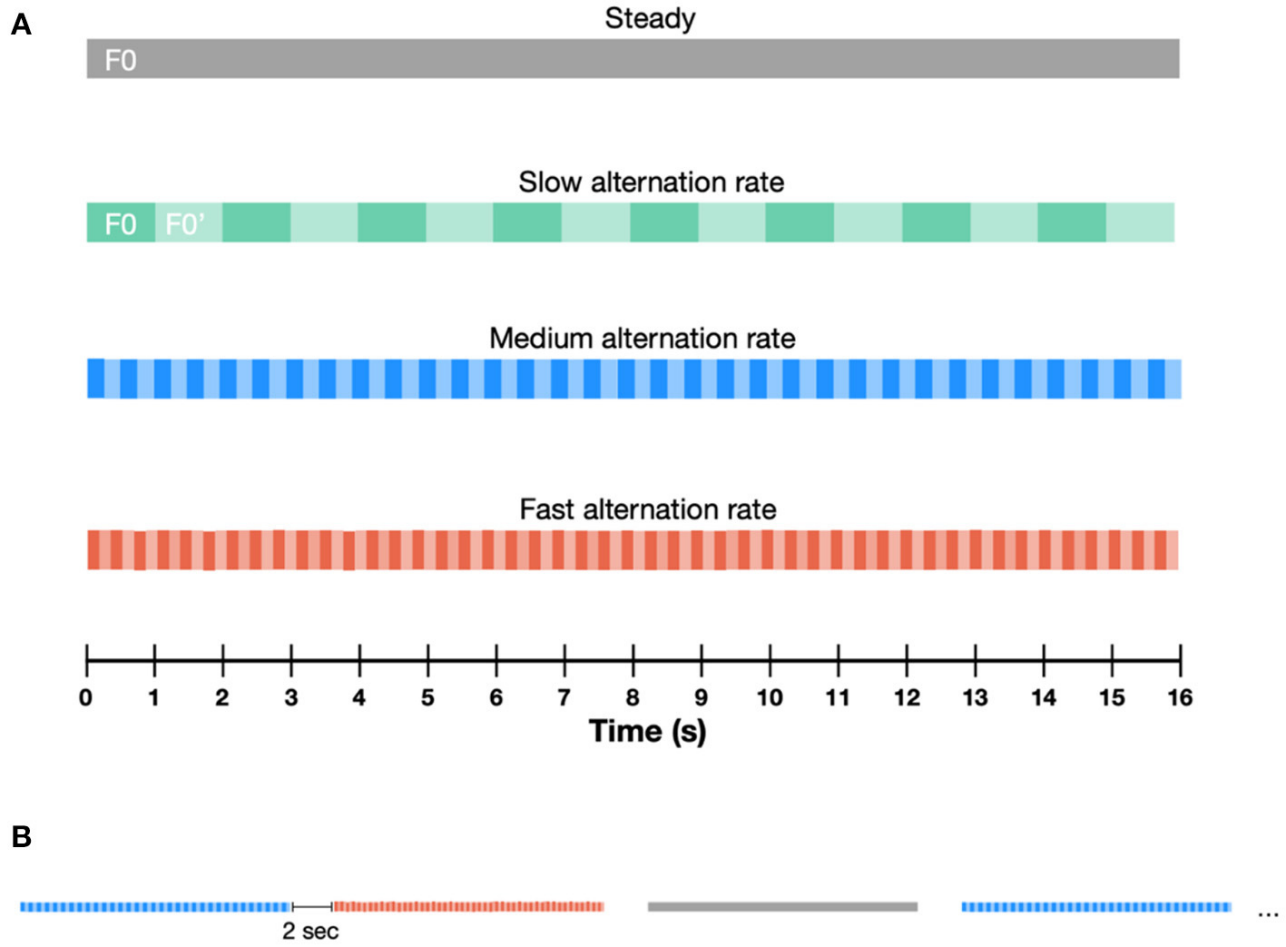
Schematic illustration of the paradigm containing different types of auditory sequences. **(A)** The F0 of these sequences was either steady or alternating between F0 and F0′, throughout the sequence. Three alternation rates were presented: slow (1 Hz), medium (2.5 Hz) and fast (6.5 Hz). **(B)** Sequence duration was fixed at 16 s. Sequences were separated by 2 s-long pauses.

Participants were presented with 16-second-long pure tone sequences. The fundamental frequency (F0) of these sequences was either steady or alternating throughout the sequence. In the steady sequences, F0 was set to 320 Hz. In the alternating sequences, both the alternation rate and the F0 varied parametrically. A schematic illustration of the paradigm is provided in Figure 1. We investigated three alternation rates (1, 2.5 and 6.5 Hz) and seven F0 changes, covering the low- (300–1,320 Hz), mid- (1,320–3,120 Hz) and high- (2,620–3,120 Hz) frequency range, with varying separations between the lower and higher F0 within each frequency range (F0 and F0′, respectively). Each F0 alternating condition consisted of two frequency pairs alternating periodically at a given alternating rate. In the low frequency range (300–1,320 Hz) F0 alternated between 320–340 Hz, 320–480 Hz, 320–720 Hz, and 320 −1,320 Hz. In the mid-frequency range (1,320–3,120 Hz), F0 alternated between 1,320–1,520 Hz and 1,320–3,120 Hz, whilst in the high-frequency range, F0 alternated between 2,620–3,120 Hz. The range of F0 were selected to cover important speech frequency range. Stimuli were presented at 75 dB (A), with alternating polarities to minimize stimulus artifacts. Sound calibration was performed separately for the low-, mid- and high-frequency ranges, as an intensity average over the whole duration of the sequences.

Sequences were presented in random order, separated by a 2 second inter-stimulus interval. Participants were presented with a total of 336 sequences (total recording time: 100 min), over two sessions that were scheduled no more than 2 weeks apart. Note that there was no significant difference in the number of rejected epochs during the first and second recording session [subcortical data: t_(9)_ = −1.58, *p* = 0.148; cortical data: t_(9)_ = −0.58, *p* = 0.574].

The number of sequences in each F0 condition was equalized across alternation rates in order to generate approximately the same number of iterations of the F0 and F0′ tones constituting sequences (see Table 1).

**Table 1:** Summary of the stimulation metrics for all three alternation rate, at one F0 change.

**Alternation**	**Number**	**Number of**	**Duration**
**rate**	**of sequences**	**F0/F0^′^ iterations**	
6.5 Hz	5	520	1.3 min
2.5 Hz	12	480	3.2 min
1 Hz	30	480	8 min

*Given that we presented seven F0 changes (see Stimuli), number of sequences and duration must be multiplied by 7 to provide total duration*.

### Recording parameters

Participants watched a muted movie with subtitles while seated comfortably in a double-walled, electrically shielded soundproof booth.

Stimuli and trigger signals were generated using a custom interface programmed in MATLAB, and delivered diotically using a external soundcard (RME FireFace UC, 44.1 kHz) connected to a custom-made trigger box which separated the two channels and simultaneously sent the trigger to the BioSemi system and the stimuli to electrically shielded ER-2 insert earphones (Intelligent Hearing Systems, Miami, FL).

Electrophysiological responses were collected using a BioSemi ActiveTwo system at a sampling rate of 8,192 Hz from 32 scalp electrodes positioned in the standard 10/20 configuration. Additional electrodes were placed on each mastoid; recordings were re-referenced offline to the average of activity at the mastoid electrodes.

### Subcortical analyses

Epochs used to analyse subcortical FFRs were obtained by applying a band-pass filter (200–4,000 Hz) to the EEG data recorded at Cz, epoching the data 0–16 s relative to target onset, and averaging across epochs. Averaged mastoids to vertex (Cz) is a commonly used electrode montage (12). The average response was transformed to the frequency-domain (FFT of 131072 points) at a resolution of 0.0625 Hz. Trials exceeding ± 100 μ at Cz or Fz were excluded, leading to an average of 2% rejected trials.

The frequency peak was computed as the highest amplitude within 1 Hz centered around the target frequencies of a given sequence. Spectral noise floor was computed as the mean amplitude within 10 Hz surrounding the target frequencies (5 Hz on each side, excluding 5 immediately adjacent bins).

### Cortical analyses

Evoked potentials of cortical origin were obtained by band-pass filtering (0.5–35 Hz) the EEG waveforms recorded at electrode C3, C4, Cz (vertex of the head), F3, F4 and Fz at 35 Hz, and creating epochs lasting −0.5 to 16 s relative to each target tone onset time. Fronto-central electrodes were chosen because they are thought to provide the most reliable estimates of both FFR and ACC measures (7, 13). Epochs were baseline corrected using the mean value from −100 to 0 ms. Trials exceeding ± 100 μ at Cz or Fz were excluded, leading to an average of 18.14% rejected trials.

To obtain the transient response, the magnitude of the auditory-evoked P1, N1 and P2 for each participants' set of data was computed as the mean amplitude in a fixed time window of 30–90, 75–150, and 150–290 ms respectively, after each alternation of frequency within every sequence type. The time windows have been selected based on visual inspection of the individual ERP responses, and are coherent with the typical latencies for each peak (14). To obtain the frequency response, data were epoched using a time window of 0 to 16 s relative to each sequence onset time.

### Statistical analyses

**Figure 2:**
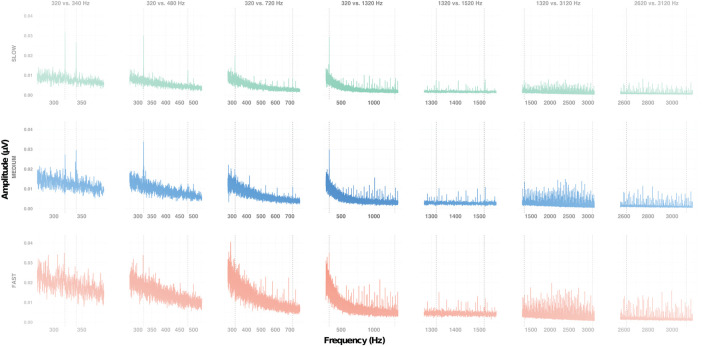
Frequency response of the subcortical grand average responses at Cz, for each condition (columns) at each alternation rate (rows). Vertical dotted lines indicate the expected frequencies for each condition.

**Figure 3:**
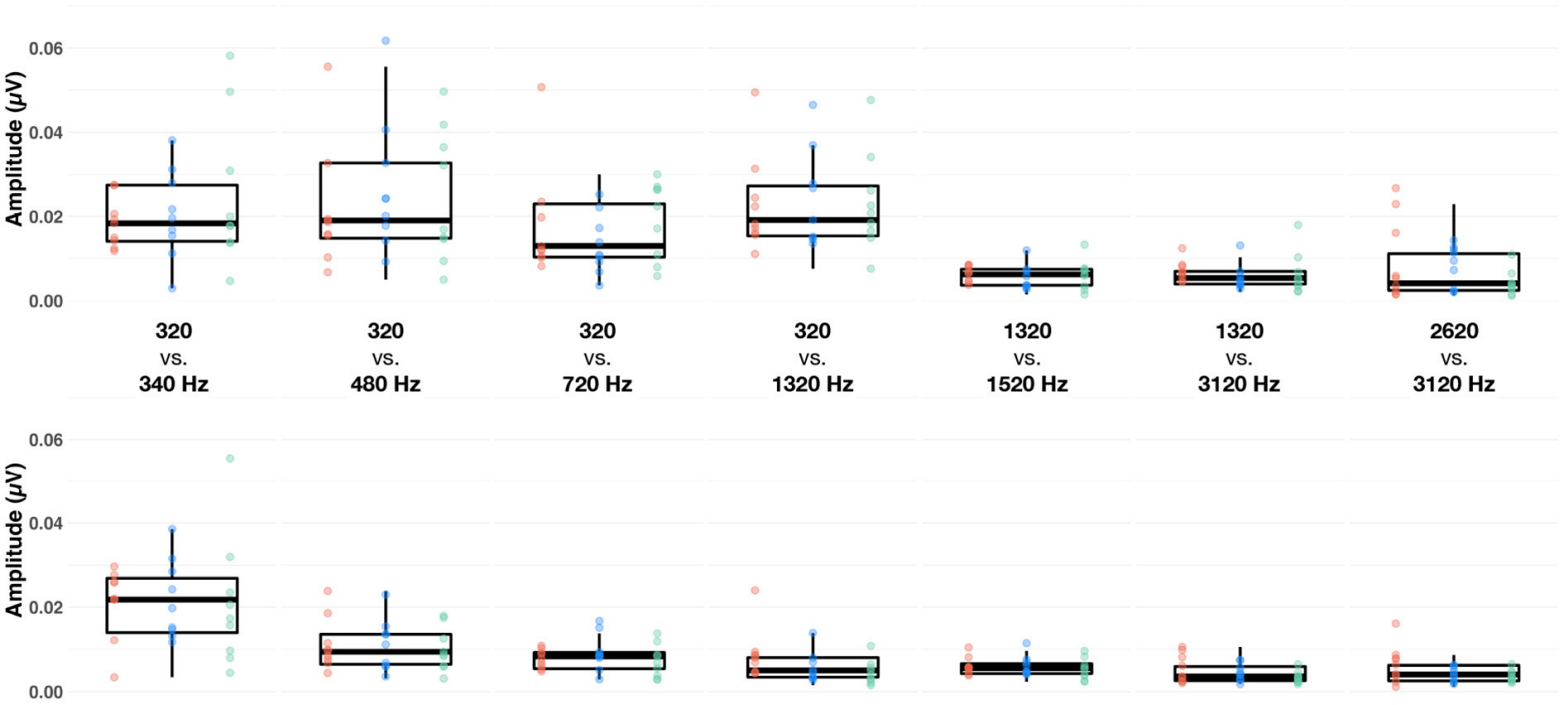
Boxplots of baseline-corrected amplitude (μV) of the subcortical response evoked by the F0 (upper row) and F0′ (lower row) in each of the seven conditions (columns), at fast (red dots), medium (blue dots) and slow (green dots) alternating rates, recorded at Cz. The whiskers indicate values that fall within 1.5 times the interquartile range. Dots falling outside the whiskers are outliers.

The aim of the first subcortical analysis was to determine whether the FFR responses were significantly above the noise floor. One outlier whose EEG responses were more than 3 S.D. above the interquartile range was excluded from the analyses of the subcortical measures, and has also been removed from the grand average plots (Figure 2) and boxplots (Figure 3). A linear mixed-effects (LME) model was used [lme4 package of R; (15)] to determine whether overall measurement type (i.e., target frequency peak or spectral noise floor), alternation rate (1, 2.5 or 6.5 Hz), condition (320 vs. 340 Hz, 320 vs. 480 Hz, 320 vs. 720 Hz, 320 vs. 1,320 Hz, 1,320 vs. 1,520 Hz, 1,320 vs. 3,120 Hz, and 2,620 vs. 3120 Hz), and F0 category (F0 or F0′), or any of their four-, three- and two-way interactions predicted the amplitude of the response. Subsequently, a LME was conducted to determine whether alternation rate, condition, and F0 category or any of their three- or two-way interactions significantly predicted the amplitude of the FFR at the target peak. In all models, the factor listener was used as a random intercept. Only the significant predictors are reported in the results section.

**Figure 4:**
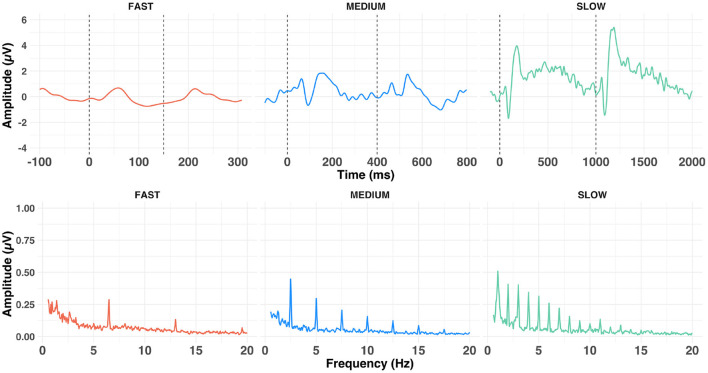
Average time- (upper row) and frequency- (lower row) domain representation of the grand average waveforms (at Cz) of the cortical response to a-ACC stimuli aggregated across conditions, presented at fast (left), medium (middle) and slow (right) alternating rates.

Visual inspection of the cortical measures (Figure 4, top panel) suggested that, as the alternation rate increased, only the P1 remained visible. This is due to the fact that, in the fast alternation condition (6.5 Hz), the ACC evoked by the new F0 started 150 ms after the previous F0, hence leading to an overlap between the P1 elicited by the new F0 and the N1-P2 of the previous sound. Therefore, statistical analyses of the cortical measures were run in two steps. First, we used an LME model to determine whether alternation rate (1, 2.5 or 6.5 Hz), condition, recording electrode and F0 range (F0 or F0′) significantly predicted the amplitude of P1. Next, we fed a LME with the same factors to determine if these could predict the N1-P2 amplitude.

The correlation between brainstem and cortical measures was investigated using Pearson correlation coefficient (r).

## Results

### Subcortical measure (FFR)

First, we set out to determine whether the amplitude of the FFR evoked by both F0 and F0′ within a sequence was significantly above the noise floor (Figure 2). The LME model including the interaction between F0 category × condition × measurement type interaction [*F* (6, 706) = 6.63, *p* < 0.001, $\eta_{p}^{2}$ = 0.05] was significant. Overall, the amplitude of the target frequency peak was always larger than amplitude of spectral noise floor, i.e., positive signal-to-noise ratio [SNR; *F*(1, 706) = 559.49, *p* < 0.001, $\eta_{p}^{2}$ = 0.44]. However, the magnitude of this effect was variable across conditions. As shown in Supplementary Figure 1, the SNR was larger for F0s in conditions 320 vs. 340 Hz, 320 vs. 480 Hz, 320 vs. 720 Hz, 320 vs. 1320 Hz than in the remaining conditions (1,320 vs. 1,520 Hz, 1,320 vs. 3,120 Hz, and 2,620 vs. 3,120 Hz). The SNR was larger for high F0s in condition 320 vs. 340 Hz than in all remaining conditions. To account for the differences in SNR in further analyses, we computed the baseline-corrected amplitude as the difference between target frequency peak and spectral noise floor (Figure 3).

Next, we sought to identify factors that influenced the amplitude of the FFR. A LME model indicated that only the factor *condition* was significant [*F*_(6,404)_ = 3.66, *p* = 0.001, $\eta_{p}^{2}$ = 0.05]. Bonferroni-corrected *post-hoc t*-tests indicated that amplitude of the FFR evoked in both 320 vs. 340 Hz and 320 vs. 480 Hz conditions was significantly larger than that evoked in the 2,620 vs. 3,120 Hz condition (both *ps* < 0.05). This suggests that, irrespective of the alternation rate, FFR amplitude is larger for low- than mid- or high- frequency range (Figure 3).

### Cortical measures

**Figure 5:**
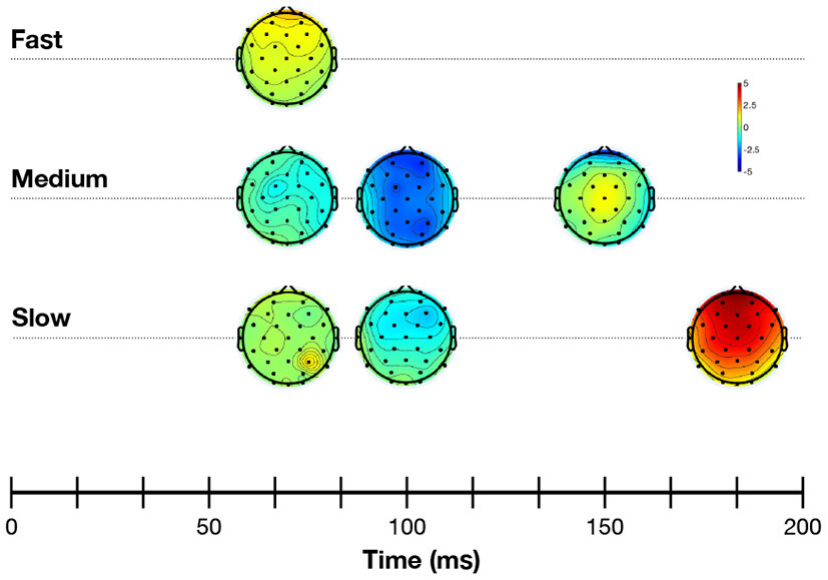
Voltage maps showing mean cortical activity during the 50–200 ms post-stimulus time window. Negative values are shown in blue, and positive values in red.

Figure 4 shows the grand average response evoked at Cz at each of the alternation rates in the time- and frequency-domain. Time-domain traces show the morphology of the response transitioned from a transient P1-N1-P2 waveform (Figure 4, slow condition) to a steady-state cortical response (Figure 4, fast condition). Voltage maps are illustrated in Figure 5. This is evident in the frequency domain plots where the spectrum transitioned from having multiple peaks (integer number of the slow and medium alternating rates) to an almost unimodal frequency peak at the fast alternating rate. Note that the morphology of the fast alternating may arise from the overlap of ACC responses leading to the steady-state sinusoidal morphology. Bearing this in mind, we will refer to P1 and N1 as the maximum and minimum of the time-domain response in the fast condition, respectively.

**Figure 6:**
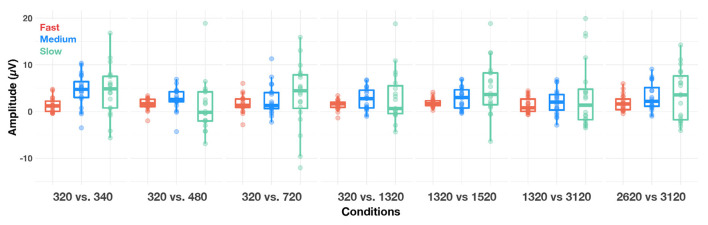
Boxplots of amplitude of the P1 (cortical) response evoked in each condition, at fast (red dots), medium (blue dots) and slow (green dots) alternating rates, recorded at Cz. The whiskers indicate values that fall within 1.5 times the interquartile range. Dots falling outside the whiskers are outliers.

An LME model applied to time-domain responses indicated that P1 amplitude was significantly affected by factors alternation rate and condition, as well as their two-way interaction [respectively: *F*_(2,2,490)_ = 41.02, *p* < 0.001, $\eta_{p}^{2}$ = 0.03; *F*_(2,2,490)_ = 5.93, *p* < 0.001, $\eta_{p}^{2}$ = 0.00; *F*_(12,2,490)_ = 3.84, *p* < 0.001, $\eta_{p}^{2}$ = 0.02]. Bonferroni-corrected *post-hoc t*-tests were used to decompose the alternation rate × condition interaction (Figure 6). P1 amplitude did not vary with condition at fast alternation rates (all *p*s > 0.10). At medium alternation rates, it was significantly larger at 320 vs. 340 Hz than any other condition (all *p*s < 0.05). At slow alternation rates, P1 amplitude was significantly smaller in 320 vs. 480 Hz than any other condition (all *p*s < 0.05). Note that overall, P1 amplitude was significantly larger at slow than medium (*p* = 0.020) alternation rate, and at medium than fast alternation rate (*p* < 0.001). This suggest that a slow alternation rate is optimal to elicit a large P1, except in the 320 vs. 480 Hz condition.

Similarly, we investigated the effect of different parameters on N1-P2 amplitudes. A LME model revealed that alternation rate, condition and EEG recording electrode, as well as the alternation rate × condition interaction were significant [respectively: *F*_(1,1,648)_ = 834.68, *p* < 0.001, $\eta_{p}^{2}$ = 0.34; *F*_(6,1,648)_ = 14.26, *p* < 0.001, $\eta_{p}^{2}$ = 0.05; *F*_(5,1,648)_ = 7.56, *p* < 0.001, $\eta_{p}^{2}$ = 0.02; *F*_(6,1,648)_ = 8.61, *p* < 0.001, $\eta_{p}^{2}$ = 0.03]. N1-P2 was significantly smaller at C3 and C4 than at F3, F4 and Fz (all *p*s < 0.05). Bonferroni-corrected *post-hoc t*-tests were used to decompose the alternation rate × condition interaction. At medium alternation rates, N1-P2 amplitude observed in conditions 320 vs. 1,320 Hz and 1,320 vs. 3,120 Hz were significantly smaller than observed in conditions 1,320 vs. 1,520 Hz and 2,620 vs. 3,120 Hz, respectively (both *p*s < 0.05). At slow alternation rates, N1-P2 amplitude was smaller in condition 320 vs. 340 Hz than in all other conditions (all ps <0.01) except in 1,320 vs. 3,120 Hz (*p* = 0.445). On the contrary, N1-P2 amplitude was larger in condition 320 vs. 480 Hz than both conditions 1,320 vs. 1,520 Hz and 1,320 vs. 3,120 Hz (both *p*s < 0.05). Last, N1-P2 amplitude was larger in condition 2,620 vs. 3,120 Hz than all other conditions (all *p*s < 0.05), except 320 vs. 480 Hz (*p* = 0.085). Note that, similarly to P1 amplitude, N1-P2 amplitude was significantly larger at slow than medium alternation rate (*p* < 0.0001). Together, this suggest that a slow alternation rate might not influence the amplitude of the subcortical response (see above), but appears to be the optimal candidate to elicit large transient cortical responses.

**Figure 7:**
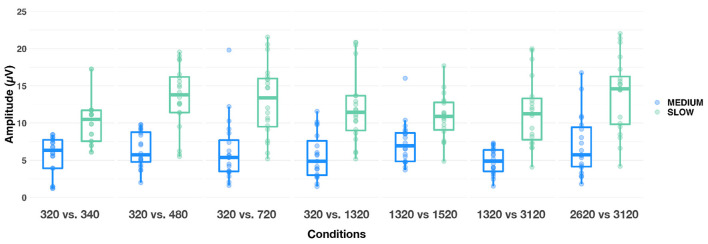
Boxplots showing N1-P2 amplitude responses evoked in each condition, at medium (blue) and slow (green) alternating rates. The whiskers indicate values that fall within 1.5 times the interquartile range. Dots falling outside the whiskers are outliers.

As an exploratory follow-up, we sought to determine whether increasing frequency separation between F0 and F0′ led to a larger N1-P2 amplitude difference. This analysis was only conducted on the four conditions where F0 = 320 Hz. A LME model revealed that alternation rate, condition and alternation rate × condition interaction were significant [respectively: *F*_(1,453)_ = 250.9, *p* < 0.001, $\eta_{p}^{2}$ = 0.36; *F*_(3,453)_ = 7.48, *p* < 0.001, $\eta_{p}^{2}$ = 0.05; *F*_(3,453)_ = 7.53, *p* < 0.001, $\eta_{p}^{2}$ = 0.05]. Bonferroni-corrected *post-hoc* comparisons failed to show significant amplitude differences between conditions at the medium alternation rate (all *p*s >0.50, see first 4 Conditions in Figure 7). However, at the slow alternation rate, the 320 vs. 480 Hz condition led to significantly larger N1-P2 amplitude differences than all three other conditions (all *p*s ≤ 0.01). N1-P2 was also significantly larger in the 320 vs. 720 Hz condition than in the 320 vs. 340 Hz (*p* < 0.01). No other comparisons were statistically significant (*p*s > 0.10).

### Relationship between subcortical and cortical measures

**Figure 8:**
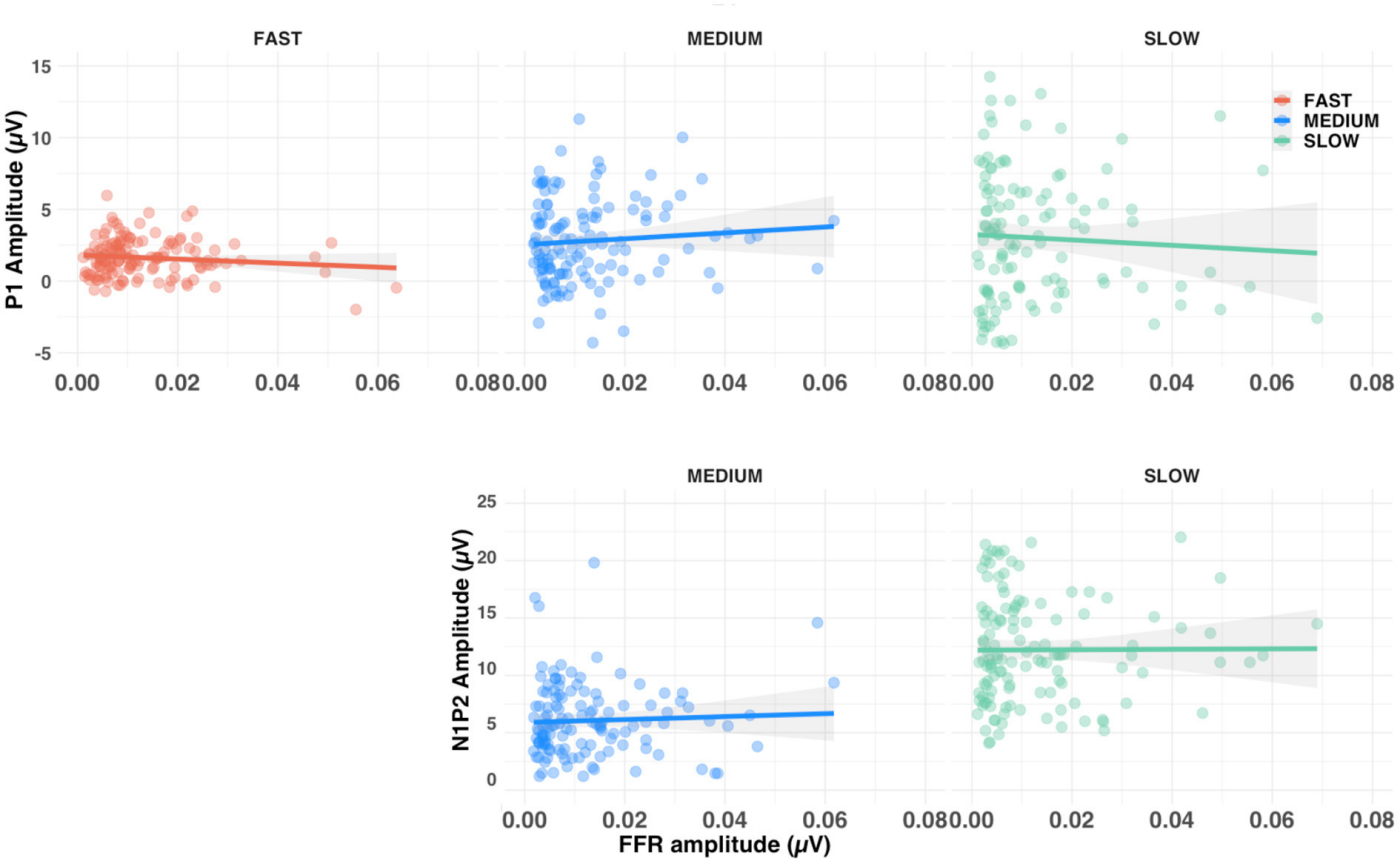
Scatterplots of the relationship between FFR amplitude and both P1 amplitude (upper row) and N1-P2 amplitude difference (lower row). Shaded lines represent the 95% confidence interval around the linear regression. Note that the fast-alternating rate did not allow enough time to elicit an N1-P2, which are not shown.

To investigate the relationship between brainstem and cortical responses we computed the correlation between P1 amplitude and FFR amplitude, as well as between N1-P2 amplitude difference and FFR amplitude. After aggregating conditions for each of the three alternation rates, none of the correlations were found to be significant (all *p*s >0.10), see Figure 8. Similarly, there was no significant correlation between the amplitude of either F0 or F0′ subcortical response and amplitude of the ACC (all *p*s >0.10).

## Discussion

The aim of this study was to identify stimulus parameters that would maximize simultaneous recording of subcortical (FFR) and cortical (OR) responses to the alternating ACC. Using this paradigm, we were able to measure significant cortical ACC and subcortical FFRs using the same stimuli. The alternating ACC maximizes data collection efficiency because each stimulus change produces a response for averaging and time is not wasted in dead periods between stimulus presentation.

The cortical and subcortical responses demonstrated different patterns across frequency range (conditions), frequency differences and alternation rate. Using a repeated-measures design (n = 10), it appears that the optimal condition for simultaneous subcortical and transient cortical recording was slowly alternating (1 Hz) between either 320 and 340 Hz or 320 and 480 Hz (see Figure 4 upper row, Figures 6, 7). Subcortical FFRs were overall larger in the low frequency range, and for F0 than F0′, consistent with more robust phase-locking at lower than higher frequencies (see Figures 2, 3). All transient cortical measures were larger at slower alternation rates, consistent with adaptation to repeating stimuli in the human auditory cortex (16). The choice of F0 conditions might depend upon the ACC response of interest. To maximize P1 amplitude, one might prefer the 320 vs. 340 Hz condition rather than 320 vs. 480 Hz condition, which elicited the smallest P1 response. However, 320 vs. 480 sequences elicited the largest N1-P2 difference. To our knowledge, this is the first study that parametrically explored auditory stimulation for optimizing recording parameters. Further studies replicating this finding on larger sample sizes would be useful both for researchers and clinicians.

A previous study investigated the use of several presentation schemes to measure the ACC to frequency changes (17). In their study, the maximum time interval between alternations was 500 ms and the reported RMS amplitudes for the ACC were in the range of 0.5 to 1 μV in adult listeners. This is smaller (roughly 3 μV if we estimate the peak-to-peak amplitude from the RMS scaling by sqrt(2) to obtain the peak amplitude and assuming that positive and negative peaks have the same peak amplitude) but comparable to our medium condition, where we observed N1-P2 amplitudes in the order of 5 μV. However, this was significantly smaller than in the slow alternating rate, where the average ACC amplitude was on average 12 μV, both of which were obtained with a similar number of epochs and presumably a similar amount of background noise. Recording time for any condition of the slow (or medium) alternating rate was 8 min, making it considerably faster than previous studies using short, broadband stimuli [e.g., (6)]. Interestingly, a similar alternation rate was successfully used to elicit electrically-evoked FFR and ACC in cochlear implant users (8, 18).

Whilst the slow alternating rate seems to be optimal for the detection of transient ACC in the time-domain, we did not investigate whether frequency-domain analysis will lead to improved detection of the ACC. A visual inspection of Figure 4 shows that use of a periodic alternation rate leads to a spectrum with peaks at the alternation rate and its harmonics. Therefore, the detection of the ACC could be performed in the frequency-domain by taking the energy of the frequency bin corresponding to the alternation rate and its harmonics and comparing those to unrelated frequencies. It remains unclear whether this approach will lead to better results than in the time-domain but it could be a promising method for detecting the ACC. Further studies could investigate if this approach can indeed improve the detection of the ACC for clinical applications.

There was no significant relationship between amplitude for subcortical and either (cortical) P1 or N1-P2 response, suggesting that they are measuring different aspects of perception. This might appear to contrast with the literature showing significant brainstem-cortical relationships (6, 19). However, previous reports showed correlations between subcortical FFR responses and late (> 500 ms), cortical pitch responses; or with N1 and P2 latency (19). Our results do not indicate a clear relationship between the subcortical FFR amplitude and cortical P1 or N1-P2 amplitudes most likely due to the different generators of the responses and the nature of their behaviors [for reviews, see (20, 21)].

We anticipate that these measures will be useful for objectively studying auditory processing in populations such as children with dyslexia or auditory processing disorders (22–25). Indeed, simultaneously acquired FFR and OR ACC would be able to inform personalized auditory training programs, enable teachers to position children in classroom locations with good signal-to-noise ratios and provide clinicians with information to optimally set up hearing aids, CIs or a combination of both.

## Conclusion

We believe that the alternating ACC paradigm can be used to measure sub-cortical and cortical responses that provide complimentary information regarding auditory processing. For probing auditory discrimination we recommend the use of slow alternation rates (<3 Hz) in the low-frequency range (300–1,200 Hz) to strike a balance between the sub-cortical and cortical levels of processing. Future work is required to evaluate how this can be used to inform clinical interventions for people with CIs or other auditory processing difficulties.

## Data availability statement

The raw data supporting the conclusions of this article will be made available by the authors, without undue reservation.

## Ethics statement

The studies involving human participants were reviewed and approved by UCL Research Ethics Committee. The participants provided their written informed consent to participate in this study.

## Author contributions

CA and VD designed experiments. CA collected and processed EEG data and drafted the manuscript. CA and UJ conducted statistical analysis. All authors contributed to the interpretation of data and to revising the manuscript. All authors contributed to the article and approved the submitted version.

## Funding

We gratefully acknowledge funding from the People Programme (Marie Curie Actions) of the European Union H2020 grant agreement no. 798093 (EAR-DNA). Debi Vickers was funded by a Medical Research Council (MRC) Senior Fellowship in Hearing (MR/S002537/1) and a National Institute Health and Care Research programme grant for applied research (201608).

## Conflict of interest

The authors declare that the research was conducted in the absence of any commercial or financial relationships that could be construed as a potential conflict of interest.

## Publisher's note

All claims expressed in this article are solely those of the authors and do not necessarily represent those of their affiliated organizations, or those of the publisher, the editors and the reviewers. Any product that may be evaluated in this article, or claim that may be made by its manufacturer, is not guaranteed or endorsed by the publisher.
