# Supplementary figures and images for: Simultaneous subcortical and cortical electrophysiological recordings of spectro-temporal processing in humans
Source: Front Neurol. 2022 Aug 3;13:928158. doi: 10.3389/fneur.2022.928158 (PMC9381701; doi:10.3389/fneur.2022.928158)

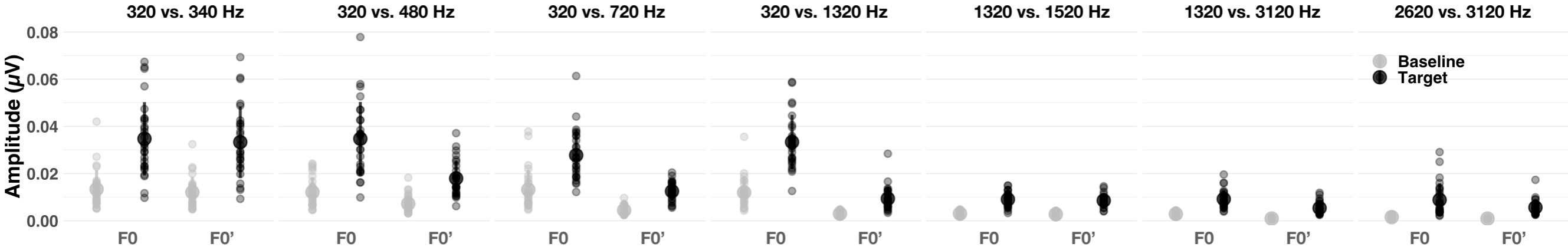

Supplement: Supplementary file 1 [file Image_1.pdf]
